# Assessment of reporting quality of abstracts of systematic reviews with meta-analysis using PRISMA-A and discordance in assessments between raters without prior experience

**DOI:** 10.1186/s12874-019-0675-2

**Published:** 2019-02-14

**Authors:** Katarina Maticic, Marina Krnic Martinic, Livia Puljak

**Affiliations:** 1 Pharmacy Mirna Jelic Hrgic, Brodski Stupnik, Croatia; 2 Department of ENT, Head and Neck Surgery, University Hospital Center, Split, Croatia; 3 Center for Evidence-Based Medicine and Health Care, Catholic University of Croatia, Ilica 242, 10000 Zagreb, Croatia

**Keywords:** Abstract, Systematic review, Reporting, PRISMA

## Abstract

**Background:**

Reporting quality of systematic reviews’ (SRs) abstracts is important because this is often the only information about a study that readers have. The aim of this study was to assess adherence of SR abstracts in the field of anesthesiology with the reporting checklist PRISMA extension for Abstracts (PRISMA-A) and to analyze to what extent will the use of PRISMA-A yield concordant ratings in two raters without prior experience with the checklist.

**Methods:**

We analyzed reporting quality of SRs with meta-analysis of randomized controlled trials of interventions published in the field of anesthesiology from 2012 to 2016 by using 12-item PRISMA-A checklist. After calibration exercise, two authors without prior experience with PRISMA-A scored the abstracts. Primary outcome was median adherence to PRISMA-A checklist. Secondary outcome was adherence to individual items of the checklist. We analyzed whether there was improvement in reporting of SR abstracts over time. Additionally, we analyzed discrepancies between the two raters in scoring individual PRISMA-A items.

**Results:**

Our search yielded 318 results, of which we included 244 SRs. Median adherence to PRISMA-A checklist was 42% (5 items of 12). The majority of analyzed SR abstracts (*N* = 148, 61%) had a total adherence score under 50%, and not a single one had adherence above 75%. Adherence to individual items was very variable, ranging from 0% for reporting SR funding, to 97% for interpreting SR findings. Overall adherence to PRISMA-A did not change over the analyzed 5 years before and after publication of PRISMA-A in 2013. Even after calibration exercise, discrepancies between the two raters were found in 275 (9.3%) out of 2928 analyzed PRISMA-A items. Cohen’s Kappa was 0.807. In the item about the description of effect there were discrepancies in 59% of the abstracts between the raters.

**Conclusion:**

Reporting quality of systematic review abstracts in the field of anesthesiology is suboptimal, and did not improve after publication of PRISMA-A checklist in 2013. We need stricter adherence to reporting checklists by authors, editors and peer-reviewers, and interventions that will help those stakeholders to improve reporting of systematic reviews. Some items of PRISMA-A checklist are difficult to score.

## Background

Usability and transparency of literature depends on its reporting quality. For this reason, many reporting checklist have been developed, for various types of studies, including study abstracts, facilitated by the EQUATOR Network (Enhancing the Quality and Transparency Of health Research) [1, 2]. Reporting quality of abstracts is particularly important because readers will often have access only to an abstract of a manuscript and even if they have access to the full text they will often decide about reading the full text depending on the information available in the abstract. Furthermore, manuscript published in languages other than English may have only abstract available in English, and then the abstract becomes the only source of information that is available to readers [3].

Systematic reviews (SRs) are considered to be the gold standard of evidence synthesis [4], and as such, quality of their reporting is of paramount importance. In 2013, the Preferred Reporting Items for Systematic Reviews and Meta-Analyses (PRISMA) extension for abstracts (PRISMA-A) checklist was published with the aim of improving reporting of SR abstracts [3]. To our best knowledge, only a few studies so far have analyzed reporting quality of SR abstracts according to the PRISMA-A that were published as full-text manuscripts in scholarly journals [5–8]. However, they were focused either on general topics [6, 8], or very specific topics i.e. depression screening tool [5] or Iranian SRs [7] and two of them included abstracts that were published before publication of PRISMA-A [7, 8].

Considering the importance of abstracts’ reporting, there was minimal effort so far invested in analyzing whether SR authors adhere to the PRISMA-A. Since pain management is considered fundamental human right [9], it could be argued that importance of proper reporting in the field of anesthesiology and pain is extremely important for both patients and health care workers. Therefore, the aim of this study was to analyze reporting quality of SRs with meta-analysis of randomized controlled trials (RCTs) of interventions published in the field of anesthesiology from 2012 to 2016, to see whether there was a temporal trend towards improvement of abstracts’ reporting quality after introduction of the PRISMA-A checklist.

## Methods

### Ethics

This study analyzed data from published studies; no patient data were included.

### Definitions

We defined SR as a study with a clear research question, systematic methods to identify, select and appraise the literature, where authors extracted and analysed/synthesized data from included studies [10]. Studies were not considered “systematic” if they had only one author or that searched only one database [11].

### Inclusion criteria

We included SRs/meta-analyses of RCTs about interventions published from 2012 to 2016 in all journals within the JCR category Anesthesiology, based on the Journal Citation Reports (JCR) impact factor for year 2016.

### Study search and screening

We searched PubMed using a filter for meta-analysis, date (years 2012–2016) and journal name. We exported search results, and screened eligibility of the abstracts. One author screened the abstracts and the second author verified the screening results.

### Exclusion criteria

We excluded diagnostic SRs, empty SRs (i.e. did not include a single study), withdrawn SRs and overviews of systematic reviews. If the SR included both randomized and non-randomized studies, it was excluded.

### Abstracts’ reporting quality

Two authors independently analyzed reporting quality of included SR abstracts against the 12 items of the (PRISMA-A). We reviewed in detail original publication that described PRISMA-A [3] and developed a coding manual. We performed a calibration exercise by two authors on a sample of ten abstracts. Both raters of abstracts have a biomedical degree, with no previous experience with PRISMA-A. We scored individual items of PRISMA-A as ‘yes’ or ‘no’, according to the coding manual. We analyzed percentage of discrepancies between the two independent authors for each item of the PRISMA-A. All discrepancies between the two authors were resolved in the consensus phase via discussion and finalized data sheet with scores was analyzed. For items with initial discrepant opinion, the final adherence status was determined by the agreed result in the consensus phase.

### Outcomes

Primary outcome was total median adherence to PRISMA-A. Secondary outcome was adherence to individual items of PRISMA-A.

### Data analysis

We made descriptive statistics using frequencies and percentages for each analyzed item. Adherence to PRISMA-A checklist for each item was shown with median and interquartile range (IQR). For analysis we used MedCalc software (MedCalc Corp., Mariakerke, Belgium).

## Results

Our search yielded 318 results, of which we included 244 SRs. We excluded 74 records because they were not systematic reviews, did not analyze interventions or did not include meta-analysis.

### Primary outcome: median adherence to PRISMA-A

Median adherence to PRISMA-A in the analyzed 244 SRs was 42% (IQR 42 to 50%), i.e. 5 PRISMA-A items (IQR 5 to 6 items). The majority of analyzed SR abstracts (*N* = 148, 61%) had a total adherence score under 50%. Only 4SRs (1.6%) had a total adherence score between 60 and 75% and not a single SR had total adherence to PRISMA-A that was above 75%. We did not see any improvement in the adherence to PRISMA-A over the analyzed 5 years; overall median adherence to all items of PRISMA-A in four of the five analyzed years was 42%, while in 2013 median adherence was 46%. Median adherence to individual items of PRISMA-A is shown in (Table 1), indicating that these medians did not change over time.

**Table 1:** Median adherence to individual items of reporting checklist PRISMA for Abstracts over time

Year	PRISMA-A item											
	1	2	3	4	5	6	7	8	9	10	11	12
2012	1	1	0	0	0	0	1	1	0	1	0	0
2013	1	1	0	0	0	0	1	1	0	1	0	0
2014	1	1	0	0	0	0	1	1	0	1	0	0
2015	1	1	0	0	0	0	1	1	0	1	0	0
2016	1	1	0	0	0	0	1	1	0	1	0	0

### Secondary outcome: proportion of SR abstracts that adhere to individual items of PRISMA-A

The lowest adherence with individual items of PRISMA-A was found for item 11 (funding), as neither one of the analyzed SR abstract indicated source of funding. Only two SR abstracts adhered to the item 12 (registration). Adherence of five items was above 80%; whereas adherence above 90% was found for only two items: 1 (title) and 10 (interpretation) (Table 2).

**Table 2:** Adherence to individual items of PRISMA-A in analyzed systematic reviews and frequency of discrepancies between two authors for each item

No.	PRISMA-A item	Description	Adherent, N (%)^a^	Non-adherent, N (%)^a^	Discrepancies in rating between raters, N (%)^a^
1	Title	Identify the report as a systematic review, meta-analysis, or both	234 (95.9)	10 (4.1)	0 (0)
2	Objectives	The research question including components such as participants, interventions, comparators, and outcomes	216 (88.5)	28 (11.5)	46 (19)
3	Eligibility criteria	Study and report characteristics used as criteria for inclusion	17 (7)	227 (93)	3 (1.2)
4	Information sources	Key databases searched and search dates	71 (29.1)	173 (70.9)	0 (0)
5	Risk of bias	Methods of assessing risk of bias	12 (4.9)	232 (95.1)	16 (6.6)
6	Included studies	Number and type of included studies and participants and relevant characteristics of studies	29 (11.9)	215 (88.1)	28 (11)
7	Synthesis of results	Results for main outcomes (benefits and harms), preferably indicating the number of studies and participants for each. If meta-analysis was done, include summary measures and confidence intervals	202 (82.8)	42 (17.2)	9 (3.7)
8	Description of the effect	Direction of the effect (i.e. which group is favoured) and size of the effect in terms meaningful to clinicians and patients	210 (86.1)	34 (13.9)	143 (59)
9	Strengths and limitations of evidence	Brief summary of strengths and limitations of evidence (e.g. inconsistency, imprecision, indirectness, or risk of bias, other supporting or conflicting evidence)	48 (19.7)	196 (80.3)	23 (9.4)
10	Interpretation	General interpretation of the results and important implications	236 (96.7)	8 (3.3)	7 (2.9)
11	Funding	Primary source of funding for the review	0 (0)	244 (100)	0 (0)
12	Registration	Registration number and registry name	2 (0.8)	242 (99.2)	0 (0)

^a^Percentages calculated from the total number of analyzed abstracts: 244

### Discrepancies between the raters

After all abstracts were checked against PRISMA-A by two authors individually, we analyzed discrepancies between the two authors. Even after calibration exercise, discrepancies were found in 275 (9.3%) out of 2928 analyzed PRISMA-A items. On average, there were 1.1 discrepant assessments per abstract. Cohen’s Kappa was 0.807. Number of discrepancies in each item is shown in Table 1. There were no discrepancies at all in items 1 (title), 4 (information sources), 11 (funding) and 12 (registration). Few discrepancies were observed for items 3 (eligibility criteria), 7 (synthesis of results) and 10 (interpretation). For three items discrepancies were observed in 6–11% of abstracts: items 5 (risk of bias), 7 (synthesis of results) and 9 (strengths and limitations of evidence). For item 2 (objectives) and particularly for item 8 (description of effect) we found high number of discrepancies between the two raters (Table 2).

## Discussion

We found that adherence of abstracts indexed as systematic reviews and meta-analysis to PRISMA-A reporting checklist in the field of anesthesiology was suboptimal, as median adherence to the checklist was 42% and there was no improvement in abstract reporting over the analyzed 5 years.

Very few studies were done so far on this topic. We found four published manuscripts in peer-reviewed literature that compared reporting in SR abstracts with PRISMA-A. One of them, published by Hopewell et al. in 2015, analyzed reporting of 103 SR abstracts presented at nine leading international conferences in 2010 and subsequently published in journals [8]. Since their sample of abstracts were published before publication of PRISMA extension for Abstracts in 2013 [3], the study of Hopewell at al. can be used to asses pre-PRISMA-A reporting status of abstracts. The authors themselves concluded their manuscript with the following words [quote]: “Our study will provide important baseline information, before publication of PRISMA for Abstracts guidelines, against which future impact can be measured.” [8].

However, Hopewell et al. did not use the 12 original items of the PRISMA-A as they are presented in the checklist. Instead, they split some of the items into more items. For example, item 4 about information sources was split into two items, one for key databases searched and one for search dates. Item 8 about description of effect was split into two items, one for benefit and one for harms [8]. Therefore, our results are not directly comparable for all PRISMA-A items. Results that are completely matching in our study and study of Hopewell et al. is that abstracts presented at nine major international biomedical conferences in 2010 also did not include information about SR funding and registration [8], and this was not different in years 2012–2016 in journals from the field of anesthesiology, as our study shows. Only two items that had adherence with PRISMA-A above 80% in the study of Hopewell et al. were item 1 (title) with 89% of adherence and sub-item 4 (number and type of included studies) with 81% of adherence [8]. In our study we had five PRISMA-A items with adherence above 80%, so this could be considered an improvement following the introduction of PRISMA-A.

Rice et al. analyzed reporting of 21 SRs with meta-analyses using adapted PRISMA-A checklist; the authors adapted the PRISMA-A because they analyzed only studies about depression screening tool accuracy, and therefore these SRs dit not analyze interventions but diagnostic test accuracy (DTA) [5]. While their results indicated that quality and completeness of reporting in the analyzed SRs were suboptimal [5], we have to emphasize that they analyzed SRs published from 2007 to 2016. Beller et al. published PRISMA-A checklist in April of 2013 [3], and if we assume some lag time needed for raising awareness of the PRISMA-A, it would be reasonable to expect that manuscripts published in 2014 or 2015 could be expected to adhere to the PRISMA-A checklist.

Rice et al. have presented detailed analysis of adapted PRISMA-A for each of their 21 included studies in their manuscript. By analyzing their data, it can be seen that even if we limit post-PRISMA-A SR abstracts to year 2015 and afterwards, this would include 5 of their 21 SRs, and median total percent of adherence to the adapted PRISMA-a was 29% before 2015 and 36% in SR abstracts published in 2015 and 2016, i.e. post-PRISMA-a. Even if we take two SRs published in 2014 as post-PRISMA-A cohort, the median total adherence is still 36% [5].

Rice et al. split item 3 and 4 into two sub-items. Their overall results indicated that items 2 (objectives), 3b (report characteristics), 11 (funding) and 12 (registration) were fulfilled in virtually no abstracts. The highest adherence in their sample of abstracts was found for reporing item 1 (title) and 10 (interpretation) [5], which was in agreement with our results. Some results were strikingly different; for example in the study of Rice et al. item 2 (objectives) was reported in 0% of abstracts, and in our study in 89% of abstracts, but this could have been due to the fact that they analyzed diagnostic test accuracy (DTA) studies and their adapted item 2 asked for “the research question including components such as participants, index test, reference standard and outcomes” [5].

Bigna et al. used PRISMA-A checklist to analyze quality of 204 abstracts of SRs with meta-analysis of RCTs published in high-impact general medicine journals in 2012, 2014 and 2015. They reported that the mean number of items reported was 7.2 in 2012, 6.8 in 2014 and 7.5 in 2015 [6]. In our sample the median number of reported numbers was 5 in all analyzed years. Bigna et al. used original 12 PRISMA-A items without adaptations. In their methods the authors indicated that they included “all systematic reviews” in the specified time frame, without specifying whether they included only SRs of interventions, and how did they score SRs of DTA and other types of SRs if they had such reviews in their sample. Bigna et al. concluded that the reporting quality of analyzed SR abstracts did not improve in 2014 and only slightly improved in 2015; they also found that reporting was better in abstracts structured with 8 headings and abstracts with word count under 300 words [6].

This finding that the reporting was better in shorter abstract is highly relevant, because it may be intuitive to consider that the quality of reporting may be hindered with restrictive word counts. The findings of Bigna et al. refer to word count under 300 words [6], but some journals, for example, require abstracts limited to 200 words, and in those circumstances it may be difficult to report all key domains suggested by the PRISMA-A. Journal editors should reconsider using very restrictive word counts in abstracts to enable proper reporting. As the authors of the PRISMA-A indicated, abstracts are not supposed to replace full-text manuscript, but for time-pressed readers or those without access to full-text reports, it is important that abstract contains relevant information [3].

Bigna et al. did not present detailed results for all analyzed abstracts in terms of adherence to individual items of PRISMA-A; instead showed those results separately for three cohorts of abstracts published in each year in Table 3 [6]. From this table one can see that the results in terms of adherence to individual PRISMA-A items were very different in some items in different years, and similar in other items; there was no uniform trend of improvement in adherence in all PRISMA-A items in analyzed abstracts over the 3 years. Results that stand out are those for items 11 (funding) with overall adherence across 3 years of 28% and 12 (registration) with a total adherence of 9.3% [6]. Adherence to those two items was much better compared to other studies, including ours.

In 2017, Kazerani et al. reported their analysis of abstracts of Iranian SRs and meta-analyses indexed in Web of Science (WoS) and Scopus published from 2003 to 2012 by using PRISMA-A [7]. However, PRISMA-A was published until 2013 [3], so the authors of the abstracts analyzed by Kazerani et al. [7] did not yet have a chance to adhere to the reporting checklist that was not available at the time the SR was published. Kazerani et al. showed that the reporting quality of 293 analyzed SR abstracts was suboptimal, with the highest adherence on item 1 (title), 2 (objectives) and 7 (synthesis of results), which was above 80% for all three. The lowest adherence was described for items 3 (eligibility criteria), 5 (risk of bias), 6 (included studies), 9 (strengths and limitations) and 12 (registration) with adherence under 6% for all those items [7].

Our data also imply that rating adherence to reporting checklists should be done by at least two raters because we had high discrepancies in few checklist items; for one item – description of the effect – we had discrepancies between two raters in as many as 59% of analyzed abstracts. On the contrary, for four items we did not have a single discrepancy, which indicates that some items of PRISMA-A are straightforward for scoring and not ambiguous at all. These four items with no discrepancies address reporting of title, information sources, funding and protocol registration. We had overall high number of discrepancies between the two raters, and the overall inter-rater agreement was high, and the majority of discrepancies were driven by differences in scoring of the two items –item #2 about objectives and item 8 about description of effect. Raters in this study were first-time raters with no prior experience with PRISMA-A, and this could have contributed to differences, but as our results indicate, discrepancies were not evenly distributed between different PRISMA-A items, which indicates that some items are more difficult to score uniformly across raters.

In the four publications that we found that used PRISMA-A for analyzing reporting of SR abstracts [6, 7], two reported that two authors analyzed abstracts independently [5, 6], one vaguely indicated that data extraction “was carried out by teams of assessors working in pairs” [8], and one did not report the method for analyzing the abstracts in terms of number of raters [7]. Only one of the four manuscripts reported overall inter-rater agreement; indicating that the agreement between reviewers on all PRISMA-A items was high (Kappa = 0.79) [6]. Without more detailed information about potential difficulties with scoring individual PRISMA-A items, we cannot know whether other authors had problems with the same items as we did in this study.

Our study is one of a number of methodological studies in the field of anesthesiology, showing deficiencies in the published literature and suboptimal reporting. We have recently showed that authors of RCTs published in top anesthesiology journals do not report using SRs to inform their study design [12]. We also found that RCT abstracts presented in four consecutive World Congresses on Pain are not properly reported and not necessarily dependable sources of information, as there are frequently discrepant results between RCT presented at a conference and published subsequently in a peer-reviewed journal [13]. As Eccleston et al. indicated in 2010, there are tools that can help authors improve quality and reporting of SRs [14]. While validity of a SR may be inherently linked with the study design and time-consuming to improve after the study is completed, improving quality of reporting of SRs should not be so demanding.

Findings of our study indicate that we need interventions aimed towards authors, editors and peer-reviewers, which will improve reporting of abstracts of systematic reviews. Authors may not be aware of reporting guidelines, but editors and peer-reviewers are gate-keepers that can insist on reporting standards as prerequisites for publication. Many journals have endorsed PRISMA statement, but PRISMA-A is extension, and therefore instructions for authors should explicitly mention not only PRISMA, but also its extensions, so that authors can comply with them.

It could be argued that suboptimal compliance with a reporting checklist for abstract is due to inadequate peer-review, and that peer-reviewers should pay more attention to the quality of reporting. However, reviewers themselves may not be aware of the existence of the relevant reporting checklist for the type of manuscript they are reviewing, and journals will rarely specifically instruct reviewers to check a manuscript against a reporting checklist. Editors could contribute to enhanced peer-review in terms of reporting quality by asking reviewers explicitly to provide feedback about the compliance of a manuscript with relevant checklists that will address both abstract and the full text.

Furthermore, it could be argued that suboptimal reporting of the abstract can be an indicator of suboptimal reporting of the entire manuscript, but we did not study this potential association in our manuscript. The original PRISMA statement was published in 2009 [15], and the PRISMA-A extension was published 4 years later. It is possible that the authors are aware of the PRISMA and use it for reporting the full manuscript, but not aware of the existence of PRISMA-A.

In this study we did not analyze whether journals mention PRISMA-A in their instructions for authors, because we analyzed manuscripts published over 5 years, and there is also lag between submission and publication, and instructions for authors may change and evolve. For this reason, current state of instructions for authors would not be informative for those past publications, but it is something that editors should consider to include explicitly in their instructions if they want better reporting.

Additionally, editors can use tools such as Penelope to help authors evaluate their manuscripts before submission, or after submission, to help them achieve better adherence with reporting guidelines [16]. Penelope or a similar information technology tool could be customized to help authors and editors improve reporting in systematic reviews.

## Conclusions

Reporting quality of analyzed abstracts of systematic reviews of interventions with meta-analysis in the field of anesthesiology was suboptimal, and it did not improve after publication of PRISMA-A checklist in 2013. We need stricter adherence to reporting checklists by authors, editors and peer-reviewers, and interventions that will help those stakeholders to improve reporting of systematic reviews.

## Abbreviations

DTA: Diagnostic test accuracy
EQUATOR Network: Enhancing the Quality and Transparency Of health Research
IQR: Interquartile range
PRISMA: Preferred Reporting Items for Systematic Reviews and Meta-Analyses
PRISMA-A: Preferred Reporting Items for Systematic Reviews and Meta-Analyses extension for abstracts
RCT: Randomized controlled trial
SR: Systematic reviews
WoS: Web of Science
